# Analysis of skeletal pain, general symptoms and patient-reported outcome measures and their value in detecting symptomatic progression – An interdisciplinary prospective study in patients with multiple myeloma^☆^

**DOI:** 10.1016/j.jbo.2025.100685

**Published:** 2025-05-08

**Authors:** Carlotta Pietsch, Monika Engelhardt, Gabriele Ihorst, Laura Wystrach, Johannes Jung, Hagen Schmal, Andreas Frodl, Ralph Wäsch, Evangelos Terpos, Georg W. Herget

**Affiliations:** a Department of Orthopedics and Trauma Surgery, Medical Center – University of Freiburg, Faculty of Medicine, Freiburg, Germany; b Comprehensive Cancer Center Freiburg (CCCF), Medical Center - University of Freiburg, Faculty of Medicine, Freiburg, Germany; c Department of Medicine, Hematology and Oncology, Medical Center – University of Freiburg, Faculty of Medicine, Freiburg, Germany; d Clinical Trials Unit, Medical Clinic, Medical Center – University of Freiburg, Faculty of Medicine, Freiburg, Germany; e Department of Orthopedics and Traumatology, Agaplesion Elisabethenstift, Darmstadt, Germany; f Department of Internal Medicine III, School of Medicine, University Hospital rechts der Isar, Technical University of Munich, Munich, Germany; g Department of Clinical Therapeutics, School of Medicine, National and Kapodistrian University of Athens, Athens, Greece

**Keywords:** Multiple myeloma, Skeletal pain, General symptoms, Clinical warning signs, Early detection of cancer, PROMs

## Abstract

Delayed diagnosis of multiple myeloma (MM) and progressive disease (PD) can both increase the risk of skeletal complications and do affects patients’ quality of life (QoL). In this prospective study we analyzed skeletal pain, general symptoms and patient-reported outcome measures (PROMs) in patients with MM and their value in detecting symptomatic progression.

We evaluated 502 patients, 47 with initial diagnosis (ID) of MM and 455 follow-up patients. At ID, 74% reported bone pain, mostly in the spine. General symptoms, particularly fatigue, were present in 89% of the patients. 88/455 (19%) of the follow-up patients experienced PD. Of these, 65% reported skeletal pain and 81% exhibited general symptoms, with fatigue being the most common. PD was suspected and confirmed as the cause of clinical symptoms in 59/88 (67%) and not suspected in 29/88 (33%). Occurrence and character of bone pain and general symptoms differed significantly between patients with and without PD, as did QoL and health-related status. Logistic regression analysis demonstrated that bone pain at night, pain in various locations, pain of known character with occurrence in different location, pain in the chest, pelvis, and thigh as well as fatigue and weight loss were associated with an increased risk of PD.

In conclusion, bone pain and general symptoms are helpful in identifying both MM and PD. PROMs can aid in the diagnosis of PD through symptom-based patient assessment. Serologic and, especially in the case of skeletal complaints, additional radiologic diagnostics are required to confirm suspected and to detect unexpected PD.

## Highlights

• Delayed diagnosis of multiple myeloma and progressive disease increase the risk of skeletal complications.
• Bone pain and general symptoms were present in most patients at initial diagnosis and progression.
• Occurrence and character of bone pain varied significantly between patients with and without progressive disease.
• Fatigue and weight loss were associated with an increased risk of PD.
• Bone pain, general symptoms and PROMs are helpful in identifying progressive disease in multiple myeloma.

## 1 Introduction

Multiple myeloma (MM) accounts for 1 % of all cancers and approximately 10 % of all hematological malignancies [1]. The majority of patients have general symptoms, most commonly fatigue [2,3]. Furthermore, more than 80 % of patients experience skeletal symptoms such as pain and pathologic fractures [4,5]. Detecting osteolysis and bone marrow lesions at the time of the MM diagnosis is therefore crucial and dictates the decision to initiate treatment [6]. In addition, MM is usually characterized by progression and relapse during the course of the disease [7,8]. Formation of new or progressive osteolysis further increases the risk for skeletal-related events (SREs).

Studies have shown that delays in diagnosing MM are associated with more unfortunate clinical courses both at initial diagnosis (ID) and in tumor progression [[9], [10], [11], [12], [13]]. Additionally, shorter overall survival and a poorer response to salvage therapy have been observed in patients with symptomatic progression [14]. Early detection of both MM and progressive disease (PD) is therefore crucial.

A recent questionnaire-based study identified several clinical symptoms evolving at the ID of MM. These warning signs may help avoid delays in diagnosis [15]. There is also evidence that incorporating patient-reported outcome measures (PROMs) into routine care is associated with increased survival compared to usual care [16].

This prospective study aimed to analyze skeletal pain, general symptoms and PROMs at initial diagnosis and in follow-up patients and their value in detecting symptomatic progression.

## 2 Material and Methods

### 2.1 Study design and patient selection

A total of 502 consecutive patients with MM were included in our prospective study. Of these, 47 (9 %) were patients with an ID of MM, and 455 (91 %) were follow-up patients seen in our comprehensive cancer center (CCC) by hematologists and orthopedics. The main inclusion criterion was a confirmed diagnosis of MM. Exclusion criteria for participation included: monoclonal gammopathy of unknown significance (MGUS), any debilitating medical or mental condition that affected a patient’s consciousness, and the inability to provide written informed consent for our thorough assessment.

Our primary objective was to analyze the occurrence and characteristics of skeletal pain and general symptoms in follow-up patients and their value – if carefully assessed by a short standardized questionnaire − in detecting symptomatic progression. Secondary objectives were to describe clinical symptoms at ID, self-reported quality of life (QoL) and health-related status, and to assess differences between patients with and without PD.

**Fig. 1:**
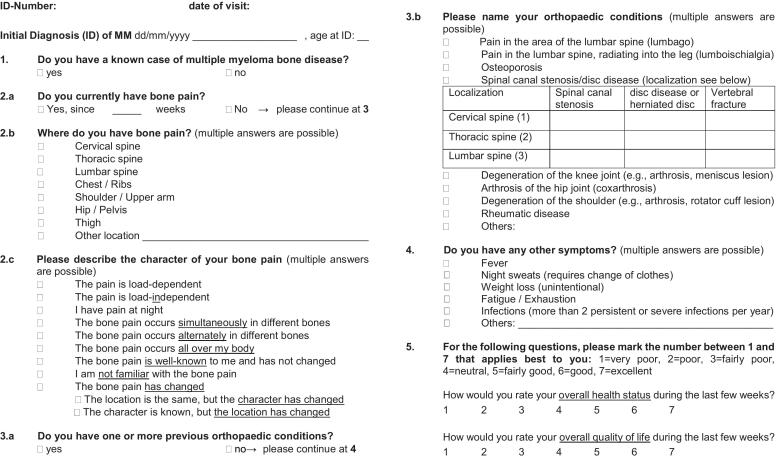
Patient questionnaire.

For the prospective interview-based analysis, we drafted a questionnaire administered to patients during their visits to the CCC outpatient clinic, either at the first contact for newly diagnosed MM or during their routine follow-up at each visit, regardless of current or previous type of treatment (Fig. 1). QoL and health-related status were indicated by patients on a simple scale from 1 to 7, with 1 = very poor and 7 = excellent. Clinical findings were correlated with serologic (performed at routine follow-up visits and in cases of clinical suspicion of PD) and radiologic diagnostics (performed in cases of clinical suspicion of PD).

The study was conducted following the Declaration of Helsinki Principles and the guidelines of Good Clinical Practice. All patients gave their written informed consent for institutional-initiated research studies and analyses of clinical outcome studies conforming to our institutional review board guidelines (amendment, Ethical committee No. 30/18).

### 2.2 Data sources for demographic and clinical information

Patient and disease characteristics were collected from electronic medical records documented in our hospital database, and our picture archiving and communication system (PACS).

### 2.3 Statistical analysis

Statistical analyses were performed using SAS version 9.4 (SAS Institute Inc., North Carolina, USA). Wilcoxon’s two-sample test was used for group comparisons of continuous data. Chi-square test was used for two-group comparisons of proportions. Multivariate logistic regression models adjusted for sex (male/female), immune globuline (IgG vs. others) and therapy, were used to assess the association between PD and clinical symptoms. Adjusted odds ratios (OR) were calculated with a 95 % confidence interval (CI). Statistical significance was defined as p-values less than 0.05. When quoting percentages, the numbers were rounded to full numbers.

## 3 Results

### 3.1 Patient’ characteristics

*All patients (n = 502):* The median age at study inclusion was 67 years (range: 41–95 years). Sixty-one percent of the patients were men and 39 % were women. Osteolytic bone lesions were detected by whole-body computed tomography (CT) in 393 (78 %) patients. Preexisting orthopedic conditions were identified in 50 % of the patients. Among these, the most common conditions were lumbar back pain and degenerative disc disease, gonarthrosis, coxarthrosis, spinal stenosis, osteoporosis and omarthrosis (Table 1).

**Table 1:** Patient characteristics.

	All patients	Initial diagnosis	Follow-up
**No. of patients**	502	47	455
**Age in years, median (range)**	64 (36–86)	65 (42–83)	64 (36–86)
**Age in years at study inclusion, median (range)**	67 (41–95)	66 (42–83)	68 (41–95)
**Sex, male/female**	61 %/39 %	45 %/55 %	62 %/38 %
**MM-type**			
IgG	53 %	49 %	54 %
IgA	14 %	21 %	13 %
Light-chain	30 %	30 %	30 %
Others (non-secretory, biclonal)	3 %	0 %	3 %
**Light-chain**			
kappa	68 %	70 %	68 %
lambda	29 %	26 %	30 %
others (non-secretory, biclonal)	3 %	4 %	2 %
**Osteolytic lesions**	78 %	81 %	78 %
Preexisting orthopedic condition(s)*	50 %	51 %	40 %
Lumbar back pain	37 %	38 %	37 %
Degenerative disc disease	36 %	46 %	35 %
Gonarthrosis	28 %	8 %	30 %
Coxarthrosis	25 %	17 %	26 %
Spinal stenosis	18 %	13 %	19 %
Osteoporosis	16 %	17 %	16 %
Omarthrosis	13 %	8 %	14 %
Rheumatoid arthritis	2 %	8 %	−

* Multiple answers allowed. The percentages of the diseases are referred to the number of patients with an orthopedic condition.

*Patients with ID of MM (n = 47)*: The median age at the time of diagnosis was 65 years (range: 42–83 years) and 66 years (range: 42–83) at study inclusion. Forty-five percent of the patients were men and 55 % were women. Osteolytic bone lesions were detected by whole-body CT in 38 (81 %) patients. Preexisting orthopedic conditions were identified in 24 (51 %) patients, including degenerative disc disease, lumbar back pain, osteoporosis, and coxarthrosis as the most common (Table 1).

*Follow-up patients (n = 455):* The median age at ID of MM was 64 years (range: 36–86 years), and age at study entry was 68 years (range: 41–95 years). Sixty-two percent were men and 38 % were women. We identified osteolytic bone lesions by whole body CT in 353 (78 %) patients. Preexisting orthopedic conditions were present in 182 (40 %) patients, and lumbar back pain, degenerative disc disease, gonarthrosis and coxarthrosis were the most common (Table 1).

### 3.2 Symptoms at ID and at follow-up

*Patients with ID of MM (n = 47)*: Skeletal pain occurred in 35 (74 %) patients. Of these, back pain was reported in 35/35 (100 %) patients and located in the cervical spine in 5/35 (14 %), the thoracic spine in 11/35 (31 %), and in the lumbar spine in 19/35 (54 %) patients. Chest pain occurred in 13/35 (37 %), pain at the shoulder/humerus in 9/35 (26 %), pain at hip(s)/pelvis in 12/35 (34 %), pain at the thigh in 3/35 (9 %), and pain in other regions in 1/35 (3 %) (Supplement Table 1**)**.

Of the 35 patients with bone pain, 18/35 (51 %) experienced motion-dependent pain, 20/35 (57 %) had pain at night, 15/35 (43 %) reported pain in various skeletal locations, and 1/35 (3 %) had pain of known character with occurrence in different location. Of note, known bone pain was reported in only 5/35 (14 %) patients, including 3 patients with a preexisting orthopedic condition and in 2 without a preexisting orthopedic condition. Additionally, 42/47 (89 %) patients exhibited general symptoms. Among these, fatigue was reported by 31/42 (74 %) patients, weight loss by 20/42 (48 %), night sweats by 9/42 (21 %), infections (more than 2 persistent or severe infections per year) by 10/42 (24 %) and fever by 1/42 (2 %) (Supplement Table 2).

*Follow-up patients (n = 455)*: Overall, bone pain was reported in 231 (51 %) patients, in 173 patients without PD, and in 58 patients with PD. Pain was predominantly located in the spine in 187/231 (81 %) patients, specifically in the cervical, thoracic, and lumbar spine in 44/187 (24 %), 42/187 (22 %), and 104/187 (56 %) patients, respectively. Pain in the chest was reported by 50/231 (22 %), shoulder pain by 59/231 (26 %), pain in the hip(s)/pelvis by 114/231 (49 %), pain in the thigh by 25/231 (11 %) and pain in other regions by 41/231 (18 %).

In the 58 patients with bone pain and confirmed PD, pain was also predominantly located in the spine in 37/58 (64 %). It was reported in the chest in 18/58 (31 %), in the shoulder in 14/58 (24 %), in the hip(s)/pelvis in 31/58 (54 %), in the thigh in 10/58 (17 %) and in other regions in 13/58 (22 %) (Supplement Table 1).

Pain was movement-dependent in 22/58 (38 %) patients, occurred at night in 34/58 (59 %), was reported at various skeletal sites in 31/58 (53 %), occurred at a known site with a different character in 8/58 (14 %), and pain of known character occurred in different location in 10/58 (17 %). The reported bone pain was known to only 15/58 (26 %) of the patients.

General symptoms in patients with PD were evident in 69/88 (78 %). Fatigue was reported by 60/88 (68 %) patients, weight loss by 28/88 (32 %), night sweats by 15/88 (17 %), and more than 2 persistent or severe infections per year by 20/88 (23 %) (Supplement Table 2).

### 3.3 Suspicion and detection of progressive disease

Based on clinical symptoms, PD was suspected in 65 of the 455 follow-up patients. Suspected progression was confirmed by serologic/radiologic diagnostics in 59/65 patients (in 27 patients by serology, in 25 patients by serology and imaging, and in 7 patients by imaging). Not suspected PD was found in 29 patients. Thus, a total of 88/455 (19 %) patients had PD, and 59/88 (67 %) had clinical PD.

Evidence of bone pain differed significantly between patients with and without PD (p = 0.0016). Detailed analysis showed significant differences for pain located in the cervical spine (p = 0.028), the thoracic spine (p = 0.0048), the chest (p = 0.0016), the hip (p = 0.014), and the thigh (p = 0.0071), respectively. No significant association to PD were found for bone pain in the shoulder and the lumbar spine. Additionally, among all follow-up patients with bone pain, pain at night (p = 0.0001) and pain of known character with occurrence in a different location (p = 0.0001) differed highly significant in patients with or without confirmed PD (Supplement Table 3).

The results of the logistic regression analysis for the follow-up patients are shown in Table 2. We observed a significant association between various bone pain characteristics and the probability of PD. Bone pain per se (OR 1.9; 95 % CI: 1.1–3.1; p = 0.0126), pain at night (OR 3.9; 95 % CI: 2.3–6.8; p < 0.0001), pain at various locations (OR 2.7; 95 % CI: 1.6–4.7; p = 0.0003), and pain of known character with occurrence in a different location (OR 13.3; 95 % CI: 2.8–62.8; p = 0.0011) were independently associated with a higher risk of PD. In addition, there was a significant association between the site of bone pain and the probability of PD. Pain in the chest (OR 2.0; 95 % CI: 1.0–3.8; p = 0.048), pain at the hip(s)/pelvis (OR 1.8; 95 % CI: 1.1–3.1; p = 0.0241), and pain in the thigh (OR 2.7; 95 % CI: 1.1–6.4; p = 0.0286) were all found to be associated with an increased risk of PD.

**Table 2:** Logistic regression analysis predicting the probability of progressive disease in follow-up patients with multiple myeloma based on characteristics and location of bone pain and general symptoms.

	Patients without PD (n = 367)	All Patients with PD (n = 88)	Odds ratio*	95 % CI	p-value
**Bone pain n (%)**	**173 (47 %)**	**58 (66 %)**	**1.9**	**1.1–3.1**	**0.0126**
dependent of movement	78 (21 %)	22 (25 %)	1.2	0.7**–**2.0	0.6108
at night	47 (13 %)	34 (39 %)	3.9	2.3**–**6.8	**<0.0001**
various locations	58 (16 %)	31 (35 %)	2.7	1.6**–**4.7	**0.0003**
character known, different location	2 (1 %)	10 (11 %)	13.3	2.8**–**62.8	**0.0011**
site known, different character	22 (6 %)	8 (9 %)	1.5	0.6**–**3.8	0.3348
known by patient	79 (22 %)	15 (17 %)	0.7	0.4**–**1.3	0.2233
spine	109 (30 %)	37 (42 %)	1.5	0.9**–**2.5	0.0999
cervical	30 (8 %)	14 (16 %)	1.8	0.9**–**3.7	0.1091
thoracical	27 (7 %)	15 (17 %)	2.0	1.0**–**4.0	0.0635
lumbar	80 (22 %)	24 (27 %)	1.2	0.7**–**2.1	0.4828
chest	32 (9 %)	18 (20 %)	2.0	1.0**–**3.8	**0.0478**
shoulder	45 (12 %)	14 (16 %)	1.1	0.6**–**2.2	0.7221
hip(s)/pelvis	83 (23 %)	31 (35 %)	1.8	1.1**–**3.1	**0.0241**
thigh	15 (4 %)	10 (11 %)	2.7	1.1**–**6.4	**0.0286**
**General symptoms n (%)**	**211 (57 %)**	**69 (78 %)**	**2.5**	**1.4–4.4**	**0.0016**
fatigue	160 (44 %)	60 (68 %)	2.8	1.7**–**4.7	**<0.0001**
weight loss	55 (15 %)	28 (32 %)	2.3	1.3**–**4.1	**0.0038**
night sweats	45 (12 %)	15 (17 %)	1.4	0.7**–**2.7	0.3476
infections	57 (16 %)	20 (23 %)	1.8	1.0**–**3.3	0.0599
fever	1 (1 %)	0 (0 %)	−	−	

CI: Confidence interval.

* Adjusted for sex (m/f), immune globuline (IgG vs. others) and therapy.

Significant differences comparing all general symptoms in confirmed versus no PD were found in all patients (p = 0.0007). Detailed analysis showed significant differences for weight loss (p = 0.0002) and fatigue (p < 0.0001). No differences were found regarding night sweats and infections. Logistic regression analysis for the follow-up patients demonstrated that fatigue (OR 2.8; 95 % CI: 1.7–4.6; p < 0.0001) and weight loss (OR 2.3; 95 % CI: 1.3–4.1; p < 0.0038) were associated with a higher risk of PD (Table 2).

### 3.4 Patient-reported outcome measures (PROMs)

The median QoL for patients at ID was 4 (neutral). It was 5 (fairly good) for follow-up patients without PD, and 4 for all patients with PD; this difference was highly significant (p < 0.0001). The median reported health-related status of patients at ID was 4. It was 5 for follow-up patients without PD, and 4 for all patients with PD; this difference was also highly significant (p < 0.0001).

No significant differences in the QoL and the health-related status were observed between patients with ID and all patients with PD (Table 3).

**Table 3:** Patient-reported outcome measures (PROMs) in all patients with multiple myeloma, at initial diagnosis, and in all patients with confirmed and no progressive disease.

PROMs	Median (range)	p-value (Wilcoxon two sample test)
**All patients (n = 502)**		
Quality of life	5 (1.0**–**7.0)	−
Health-related Status	5 (1.0**–**7.0)	−
**ID of MM (n = 47)**		**ID vs. PD**
Quality of life	**4** (1.0**–**7.0)	p = 0.5635
Health-related status	**4** (1.0**–**7.0)	p = 0.7493
**Follow-up patients (n = 455)**		**No PD vs. PD**
Quality of life (no PD/PD)	**5** (1.0**–**7.0)/**4** (1.0**–**7.0)	p < 0.0001
Health-related status (no PD/PD)	**5** (2.0**–**7.0)/**4** (1.0**–**7.0)	p < 0.0001

ID: initial diagnosis. PD: progressive disease. MM: multiple myeloma. Quality of life and health-related status defined by patients: 1 = very poor, 2 = poor, 3 = fairly poor, 4 = neutral, 5 = fairly good, 6 = good, 7 = excellent.

## 4 Discussion

A delay in diagnosing MM is associated with more unfortunate clinical courses, both at ID diagnosis and in tumor progression. Understanding the characteristics of skeletal pain, along with associated general symptoms, will help in identifying MM as the underlying cause and in detecting PD in patients undergoing therapy or regular clinical supervision. Moreover, collecting PROMs could enhance the diagnosis of PD.

The main clinical manifestations of MM include elevated serum calcium, renal insufficiency, anemia and bone disease [[17], [18], [19]]. Bone disease is present in approximately 80 % of newly diagnosed MM [[17], [18], [19]], and up to 90 % of MM patients develop osteolytic lesions during the disease course [20,21]. These patients face a high risk for SREs, which contribute to the disease burden in terms of both survival and QoL [22]. MM patients who presented a fracture at the time of diagnosis revealed a lower survival rate compared to those without a fracture, and patients who experienced a fracture during the disease course had twice the risk of death compared to those who did not suffer a fracture [10]. In addition, MM patients with clinical progression had worse survival than those presenting biochemical progression [12].

This highlights the importance of early detection of MM, and the identification of PD in follow-up patients. Taking a thorough patient history is therefore essential [11].

### 4.1 Symptoms at ID of MM and in progressive disease

In the present study, the majority of the patients at initial diagnosis as well as those with progressive disease experienced bone pain, with the spine being the main site. Pain was also commonly reported in the chest, the hip(s)/pelvis, the shoulder, and the thigh. Of note, location of pain in the thoracic spine, the chest and the thigh is uncommon for an underlying (degenerative) orthopedic condition, which is typically related to joints or the more mobile and/or increased weight-bearing parts of the spine [15,[23], [24], [25]]. Furthermore, bone pain at night and pain at various locations was frequently reported, also being atypical for degenerative diseases. It is also noteworthy that bone pain was known to only 14 % of the patients with skeletal pain at initial diagnosis, regardless of whether they had an orthopedic condition. In the follow-up group, one-third of patients were able to differentiate the bone pain from their underlying preexisting orthopedic condition.

A previous study yielded similar results in patients at initial diagnosis: the most common complaint was back pain and about one-third reported pain in the chest. Pain occurred at night in about two-thirds, and was present at multiple sites in about one-third of the patients [15]. Miseer et al. reported back pain as the most common single self-reported symptom [24]. Approximately half of the patients in another study were symptomatic at ID with either bone pain and/or SREs. Of these, 47.5 % had a baseline bone pain record, predominantly affecting the back [26]. Kyle et al. reported bone pain at the MM diagnosis in 58 % of their patients, typically triggered by movement [3].

Besides bone pain, the vast majority of patients at initial diagnosis and progressive disease experienced general symptoms, with fatigue being the most common. Other frequent symptoms included weight loss and night sweats.

In one of our previous studies, fatigue, anemia symptoms, night sweats and weight loss were found to be also common symptoms at initial diagnosis [15]. Talamo et al. reported anemia in 58 % of patients at presentation [27]. Kyle et al. reported in their study fatigue in 32 % of patients, while 24 % of patients experienced weight loss [3].

To the best of our knowledge, only one larger (retrospective) study to date has described symptoms in PD. Its authors found that various CRAB symptoms coincided with the time of relapse, with new or evolving bone disease (80.9 %), followed by anemia (38 %), and renal failure (12.7 %) [12].

In summary, skeletal pain was one of the most important symptom at the ID of MM and in patients with symptomatic progression. It was most often accompanied by fatigue.

### 4.2 Suspicion and detection of progressive disease

PD was suspected in 65 and not suspected in 29 of the 455 follow-up patients. Clinical progression was confirmed by serologic/radiologic diagnostics in 59/65 patients. Thus, a total of 88 (19 %) patients had PD, and 59 (67 %) of these had clinical PD. In a large study of patients with relapsed MM, the authors found that most progressions were biochemical progressions (60.4 %), while 39.6 % had clinical progression [12]. A possible explanation for the distribution of biochemical and clinical progression in our study is that outpatient visits included a comprehensive history that encompassed both general and specific bone symptoms. The inquiry into these symptoms is somewhat time-consuming, which may impede its consistent implementation in routine clinical practice. Nevertheless, based on our results and with the help of a short standardized questionnaire, this approach could be very helpful.

Beyond the occurrence of bone pain per se, we observed significant differences in several characteristics of bone pain and in the occurrence of general symptoms when comparing patients with confirmed PD to those without. Our logistic regressions analysis revealed that pain at night, at various locations, pain with known character but at a different site, fatigue, and weight loss were independently associated with a higher risk of PD. Analysis also demonstrated that pain at the chest, hip(s)/pelvis and thigh were also independently associated with a higher risk of PD.

These findings emphasize the importance of collecting clinical data and symptoms, such as bone pain and its characteristics, as well as general symptoms during the outpatient visit to detect MM progression.

Awareness of symptoms can also alert patients that they may be the underlying cause of PD. This could motivate patients to seek medical attention prior to a routine consultation, as encouraged by us and various myeloma centers worldwide. That more frequent and sensitive monitoring of the disease has led to earlier detection of PD and earlier therapeutic intervention has been described by many, also by us in Engelhardt et al. [28].

Certainly, serologic and, especially in the case of skeletal complaints, radiologic examinations are essential to confirm the clinical suspicion of PD and to detect unexpected progression.

### 4.3 Patient-reported outcome measures (PROMs)

Incorporating PROMs within routine care is known to be associated with longer survival compared to usual care [16]. And, although many patient-reported outcomes (PROs) questionnaires were initially developed for use in clinical trials, there is rapidly growing interest to integrate PROs into routine clinical practice for monitoring patient clinical status [29].

Our study applied a very simple scale to assess both QoL and health-related status, which is practical in clinical settings where time is often limited. We identified a highly significant difference in reported QoL and health-related status between patients with and without PD, while no significant differences were found between patients at their initial MM diagnosis and patients with PD. A study on QoL in relapsed or refractory MM also revealed that most patients reported severe symptoms and poor QoL [30]. Another study on patients with relapsed and/or refractory MM also reported poor QoL, which worsened with each additional relapse [31].

A limitation of our simple approach may be the lack of sensitivity to detect subtle changes in patient status and the lack of use of validated questionnaires such as the EORTC-QLQ-C30 or MY20. However, our standardized questionnaire allows for a more thorough history of skeletal pain, general symptoms, possible changes, and an assessment of patient comfort than is often obtained in routine clinic follow-ups. Moreover, the questionnaire was very helpful in decision-making – in conjunction with laboratory results – i.e. when to perform further diagnostics like imaging, and when to seek multidisciplinary advice within our MM-tumorboard.

Further research is necessary to validate our findings in even larger, multi-centered approaches with more diverse populations (i.e. early and late myeloma relapses and those with very novel therapy approaches, such as antibody drug-conjugates (ADCs), bispecific T-cell engagers (BiTEs) and chimeric antigen receptor T-cells (CAR-T-cells)), and to explore the potential of using PROMs to personalize treatment decisions. In addition, the use of more comprehensive instruments in future research, such as the integration of PROMs into electronic health record systems, may facilitate patient-reported outcome surveys to detect symptoms early and alert clinicians [32].

## 5 Conclusion

Most patients at initial diagnosis of MM and those with progression reported bone pain and general symptoms. Bone pain at night, pain in various locations, pain of known character with occurrence in different location, pain in the chest, pelvis, and the thigh as well as fatigue and weight loss were associated with an increased risk of progressive disease. Moreover, PROMs differed significantly between patients with and without progression, highlighting their value in identifying PD.

Therefore, the systematic assessment of clinical symptoms remains crucial, both for the initial detection of MM and for monitoring disease progression. The use of a standardized questionnaire, as implemented in this study, greatly facilitates symptom evaluation. Serologic and, in the case of skeletal complaints, radiologic examinations should be regularly conducted during routine follow-up visits and whenever there is clinical suspicion of PD. This practice ensures not only objective confirmation of clinically suspected progression but also the early detection of asymptomatic and/or unexpected PD.

## 6 Disclosures

Dr. Ihorst and Dr. Schmal have nothing to disclose. Dr. Herget, Dr. Pietsch and Dr. Wystrach report grants from Janssen-Cilag. Dr. Jung reports grants from the Else Kroener Fresenius Stiftung, outside the submitted work. Dr. Wäsch reports honoraria from Pfizer, Amgen, Novartis, Sanofi, Janssen, BMS, and Kite/Gilead, outside the submitted work. Dr. Engelhardt reports grants and personal fees from Amgen, Janssen, BMS, Takeda, outside the submitted work. Dr. Terpos reports honoraria from BMS, Takeda, Menarini/Stemline, Janssen, GSK, EUSA Pharma, ASTRA/Zeneca, Amgen, Pfizer, Sanofi and travel expenses from Takeda, EUSA Pharma, Astra/Zeneca, Amgen, and Sanofi outside the submitted work.

## CRediT authorship contribution statement

**Carlotta Pietsch:** Writing – review & editing, Writing – original draft, Formal analysis, Data curation, Conceptualization. **Monika Engelhardt:** Writing – review & editing, Validation, Supervision, Methodology, Formal analysis, Data curation, Conceptualization. **Gabriele Ihorst:** Writing – review & editing, Validation, Software, Formal analysis, Data curation. **Laura Wystrach:** Writing – review & editing, Methodology, Funding acquisition, Data curation, Conceptualization. **Johannes Jung:** Writing – review & editing, Investigation, Data curation. **Hagen Schmal:** Writing – review & editing, Methodology, Formal analysis. **Andreas Frodl:** Writing – review & editing, Formal analysis. **Ralph Wäsch:** Writing – review & editing, Formal analysis. **Evangelos Terpos:** Writing – review & editing. **Georg W. Herget:** Writing – review & editing, Writing – original draft, Validation, Project administration, Methodology, Investigation, Funding acquisition, Formal analysis, Data curation, Conceptualization.

## Ethics approval and consent to participate

Approval for this study was obtained from the local ethics committee of the Albert-Ludwigs-University Freiburg (EK 30/18).

## Funding

This work was supported by Janssen-Cilag GmbH, Johnson & Johnson-Platz 1, D-41470 Neuss.

We acknowledge support by the Open Access Publication Fund of the University of Freiburg.

## Declaration of competing interest

The authors declare that they have no known competing financial interests or personal relationships that could have appeared to influence the work reported in this paper.
