# Supplementary material for: Analysis of skeletal pain, general symptoms and patient-reported outcome measures and their value in detecting symptomatic progression – An interdisciplinary prospective study in patients with multiple myeloma
Source: J Bone Oncol. 2025 May 8;52:100685. doi: 10.1016/j.jbo.2025.100685 (PMC12138919; doi:10.1016/j.jbo.2025.100685)
Supplement: Supplementary Data 1 [file mmc1.docx]

**Supplement Table 1**. Location of bone pain at initial diagnosis of multiple myeloma, in follow-up patients and in all patients with progressive disease

| Bone pain* | Initial diagnosis of MM (n=47) | Follow-up patients (n=455) |
| --- | --- | --- |
| No. of patients n (%) | 35 (74%) | 231 (51%) |
| Spine | 35 (100%) | 187 (81%) |
| cervical | 5 (14%) | 44 (24%) |
| thoracical | 11 (31%) | 42 (22%) |
| lumbar | 19 (54%) | 104 (56%) |
| Chest | 13 (37%) | 50 (22%) |
| Shoulder | 9 (26%) | 59 (26%) |
| Hip(s) / pelvis | 12 (34%) | 114 (49%) |
| Thigh | 3 (9%) | 25 (11%) |
| Others** | 1 (3%) | 41 (18%) |
|  |  |  |
| No. of patients with bone pain* and progressive disease n (%) | **-** | **58/231 (25%)** |
| Spine | - | 37 (64%) |
| cervical | - | 14 (38%) |
| thoracical | - | 15 (41%) |
| lumbar | - | 14 (38%) |
| Chest | - | 18 (31%) |
| Shoulder | - | 14 (24%) |
| Hip(s)/pelvis | - | 31 (54%) |
| Thigh | - | 10 (17%) |
| Others** | - | 13 (22%) |
| MM: Multiple myeloma; No: number of patient(s). *One or more regions of bone pain were possible in each patient. ** Others: Skull, jaw, sacrum, knee, lower leg, heel, feet, elbow, hand. The percentage number refers to the number of patients with bone pain. | | |

**Supplement Table 2.** Clinical symptoms in patients with initial diagnosis of multiple myeloma and in patients with progressive disease

|  | Initial diagnosis of MM (n=47) | All patients with PD (n=88) |
| --- | --- | --- |
| Bone pain* n (%) | **35 (74%)** | **58 (66%)** |
| dependent of movement** | 18 (51%) | 22 (38%) |
| at night | 20 (57%) | 34 (59%) |
| various locations | 15 (43%) | 31 (53%) |
| character known, different location | 1 (3%) | 10 (17%) |
| site known, different character | 5 (14%) | 8 (14%) |
| known by patient | 5 (14%) | 15 (26%) |
| with preexisting condition(s) | 3/17 (17%) | 12/34 (35%) |
| no preexisting condition(s) | 2/18 (11%) | 3/24 (13%) |
| General symptoms* n (%) | **42 (89%)** | **69 (78%)** |
| fatigue | 31 (74%) | 60 (68%) |
| weight loss | 20 (48%) | 28 (32%) |
| night sweat | 9 (21%) | 15 (17%) |
| Infections | 10 (24%) | 20 (23%) |
| fever | 1 (2%) | 0 (0%) |
| MM: Multiple myeloma. PD: progressive disease. *Multiple answers allowed. ** some patients data are missing. *** assigned to the symptoms for better presentation | | |
|  | | |

**Supplement Table 3.** Significance of bone pain characteristics in patients with bone pain with confirmed versus no progressive disease (PD)

| Bone pain characteristics | Patients with bone pain (n=231/455) | | p-value (Fisher’s exact Test) |
| --- | --- | --- | --- |
|  | confirmed PD (n=58) | without PD (n=173) |  |
| Dependent of movement n (%) | 22 (38%) | 78 (45%) | n.s. (p=0.36) |
| At night | 34 (59%) | 47 (27%) | 0.0001 |
| In various locations | 31 (53%) | 58 (34%) | 0.0082 |
| known by character, different location | 10 (17%) | 2 (1%) | 0.0001 |
| Site known, different character | 8 (14%) | 22 (13%) | n.s. (p=0.82) |
| Known pain | 15 (26%) | 79 (46%) | 0.0087 |
